# Obituary

**Published:** 1879-04

**Authors:** 


### OBITUARY.

Died, suddenly, in Philadelphia, on Monday evening March 3, 1879, Dr. John H. McQuillen, aet. fifty-three years. He was a native of Philadelphia, was educated in the Friends' School in that city, after which he entered upon commercial pursuits, which he after a time abandoned to take up the study of dentistry and medicine.

In 1849 he began the practice of dentistry; in 1852 he graduated from the Jefferson Medical College. In 1857 he was appointed to the chair of operative dentistry, in the Pennsylvania Dental College. This he accepted and occupied till 1862, when he resigned and at once set about the organization of the Philadelphia Dental College. Upon application the Legislature granted a charter, and in 1863 the college was established with Dr. McQuillen at its head, and he was its leader. It has held its regular annual sessions ever since, and through the energy and ability of Dr. McQ. it attained remarkable success, having larger classes than ever were brought together by any similar institution, except one. The college in his passing away has probably sustained an irreparable loss.

In 1869 he became editor of the Dental Cosmos, and well sustained his position, so long as he held the connection.

His writings elicited marked attention wherever they found readers, and this was almost co-extensive with the profession in the world.

He was a prominent worker in dental associations, always taking an active part in whatever he conceived to be right. He was one of the organizers of the American Dental Association, and ever manifested a jealous care for its interests.

His interest and study extended to almost every field of scientific research, but all this he made tributary to his chosen profession.

The loss to the profession is very great. He will be greatly missed by his friends and acquaintances. He leaves a widow, one daughter and three sons, who will receive a large measure of warm and sincere sympathy from all who knew the husband and father. May they be sustained and strengthened in this hour of their great sorrow.

At a special meeting of the Alumni Association of the Philadelphia Dental College, a committee was appointed on resolutions, and respectfully submit the following:

It is our painful duty to record the death of our teacher and fellow member, Prof. J. H. McQuillen,

*Resolved*, That in his death the Alumni Association of the Philadelphia Dental College has lost a friend, who was ever ready to devote his time and talents to the best interests of our Alma Mater.

*Resolved*, That in his death, the profession has lost an invaluable member, and a most earnest worker in the cause of dental education. To his indomitable energy and perseverance we bear our testimony, and mourn his departure.

*Resolved*, That a copy of these resolutions be forwarded to his bereaved family, and to the dental journals for publication.

Dr. A. Boice,
Dr. J. McManus,
Dr. A. Tees,
Dr. L. A. Faught,
Dr. W. H. Fundenberg,
Dr. J. L. Eisenbrey,
Committee.

